# Rate control strategies for atrial fibrillation

**DOI:** 10.1080/07853890.2021.1930137

**Published:** 2021-05-25

**Authors:** Muath Alobaida, Abdullah Alrumayh

**Affiliations:** Department of Basic Sciences, Prince Sultan bin Abdulaziz College for Emergency Medical Services, King Saud University, Riyadh, Kingdom of Saudi Arabia

**Keywords:** Atrial fibrillation, Rate control strategy, Electrophysiology, arrhythmias

## Abstract

Atrial fibrillation (AF) is one of the main cardiac arrhythmias associated with higher risk of cardiovascular morbidity and mortality. AF can cause adverse symptoms and reduced quality of life. One of the strategies for the management of AF is rate control, which can modulate ventricle rate, alleviate adverse associated symptoms and improve the quality of life. As primary management of AF through rate control or rhythm is a topic under debate, the purpose of this review is to explore the rationale for the rate control approach in managing AF by considering the guidelines, recommendations and determinants for the choice of rate control drugs, including beta blockers, digoxin and non- dihydropyridine calcium channel blockers for patients with AF and other comorbidities and atrioventricular nodal ablation and pacing. Despite the limitations of rate control treatment, which may not be effective in preventing disease progression or in reducing symptoms in highly symptomatic patients, it is widely used for almost all patients with atrial fibrillation. Although rate control is one of the first line management of all patient with atrial fibrillation, several issues remain debateable.

## Introduction

Atrial fibrillation (AF) is the most common cardiac arrhythmia in adults [1]. It can increase the risk of stroke, heart failure (HF) and mortality [2]. The risk of developing AF increases progressively with age [3], from a prevalence of 0.1% in individuals under 55 years of age to 9.0% in individuals 80 years and older [4], and in adults, the current prevalence of AF is 2–4% [1], with estimated rise of 2.3 folds [5] due to extended longevity in general population [6]. The exact reasons for why age and certain medical disorders increase the risk of AF are not yet fully understood [7]. Although age is an independent risk factor for adverse AF outcomes [3], the increased burden of comorbidities (i.e. coronary heart disease, heart failure, diabetes mellitus, hypertension and obstructive sleep apnoea) are significant contributors to the development and progression of AF, as well as significant modifiable risk factors [8–10].It is likely that structural (e.g. dilatation and fibrosis) and electrophysiological changes of atrial myocytes, likely disturb the electrical substrate which causes AF [6],although the mechanisms vary among patients with AF. The main aim of AF treatment is to simply follow the ABC pathway, avoid strokes, better control symptoms, and reduce cardiovascular and comorbidity risks [2]. Another important aim is to recognize and manage the associated factors, whether acute (e.g. as a result of cardiac surgery and inflammatory diseases) or chronic (e.g. as a result of heart valve stenosis, coronary artery disease, hypertension or obesity) [11]. Rate control and rhythm control treatments are the two main strategies currently in use. The rate control is defined as use of any combination of β‐blocker, non- dihydropyridine calcium channel blocker, and digoxin or AV nodal ablation without aiming to restore normal sinus rhythm. Rate control is an essential part of AF management. The goal of rate control treatment is to modulate the ventricular rate, with the aim of better symptom control, the reductions in thromboembolic, cardiovascular and comorbidity risks.

The rhythm control strategy, which could involve pharmacological intervention, is relatively effective for the maintenance of sinus rhythm, but it can have adverse drug reactions. It reduces AF recurrences, but may not always eliminate them [12], the risk of serious adverse events in patients appears to be greater, compared to rate control strategies [13].

The AFFIRM trial [14] was a comparative study of rhythm versus rate control strategies in AF patients over five years. This demonstrated no significant difference in neither the ischemic stroke rate (7.1% vs. 5.5%; respectively; *p* = .79) or mortality (23.8% vs. 21.3%; respectively; *p* = .08). Similarly, meta-analyses of a few RCTs demonstrated no significant difference with regards to both stroke-related and general mortality, in spite of bias towards rate control [15]. Thus far, comparison of rhythm vs rate control as the superior strategy for AF management remains a controversial area in the literature; however, there is still little of evidence in the management of older patients. The superiority of rate control over rhythm control was evident in terms of cost-effectiveness [16], whereas rhythm control has demonstrated better outcomes in factors such as rates of stroke/TIA and health related QoL [17,18]. In spite of this, studies from the real world demonstrate that rate control strategies are used preferentially, and especially in the management of older patients [19], while rhythm control strategies are used primarily to reduce AF-related symptoms [20].

**Figure 1.**
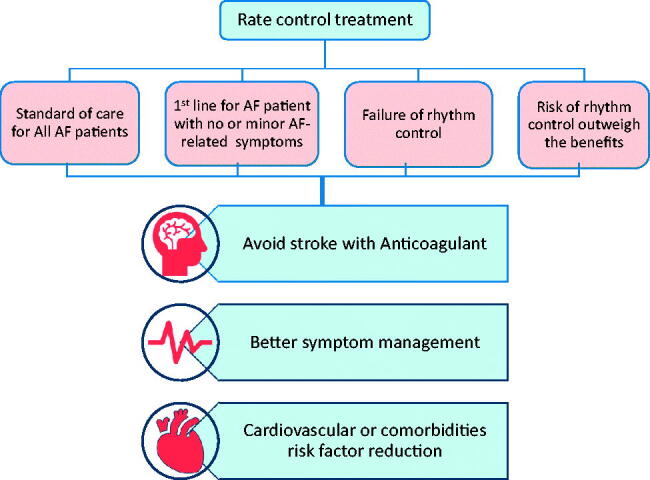
Indications of rate control treatment with the ultimate goal. AF: Atrial fibrillation [2].

This can be achieved through the rate control approach, which is easier to establish and manage, and has a lower rate of hospitalization as well as major adverse events. The purpose of this review is to explore the rationale for employing the rate control approach, by considering the guidelines, recommendations and determinants for the choice of rate control drugs and atrioventricular nodal ablations. In spite of the limitations of rate control treatment, which may not be effective in preventing disease progression or in reducing symptoms insusceptible patients, it is still widely used for almost all AF patients (Figure 1).

### The value of rate control drugs for management of atrial fibrillation

During AF, stroke volume and ventricle filling time are reduced as a result of fast and irregular ventricular rates [21]. Consequently, a reduction in the cardiac output by 20–30% [22] and irregular rhythm cause symptomatic consequences and contribute to the development or worsening of heart conditions, such as HF [23]. The dramatic increase in the heart rate also leads to substantial negative inotropic effects [21]. Patients with HF in whom the left ventricle ejection fraction (LVEF) is preserved or reduced are prone to more significant deterioration if they have AF [24]. In a nationwide large-scale AF cohort study [25], it was demonstrated that patients who received rate control treatment agents such as beta blockers (BB) or non-dihydropyridine calcium channel blockers (NDCC) had a lower mortality rate than patients who did not receive these treatments. In addition, the use of BB was associated with the lowest risk of mortality (adjusted HR: 0.76; 95% CI: 0.74–0.78), while digoxin usage was associated with the highest mortality risk (adjusted HR: 1.12; 95% CI: 1.10–1.14) [25].

Rate control therapy is preferred in three situations. First, although patients with new-onset AF (less than 1 year after diagnosis) and cardiovascular conditions are more preferred to use the rhythm control management (catheter ablation and/or pharmacological) compared to the usual care (rate control) [26]; rate control remains the mainstay treatment for almost all patients with AF. This is because maintaining well-controlled ventricular rates during AF deterioration is of the utmost importance. Second, it is the drug of choice for those who do not require rhythm control, that is, those older than 80 years of age who are asymptomatic or have mild symptoms [2,27]. Currently, the main aim of using rhythm control treatment is to improve AF-related symptoms. Third, it is the only alternative treatment used if rhythm strategy (e.g. pharmacological and catheter ablation) is unsuccessful or if the risks outweigh the benefits (e.g. bradycardia-tachycardia syndrome and high pacing risk). Therefore, it would be reasonable to consider rate control strategies in these situations, but the choice of treatment should be comprehensive and tailored to the severity of the symptoms, ventricular rates, associated co-morbidities and shared decision-making strategy [28,29].

### Rate control definition and clinical guideline recommendations

Improvement of symptoms, preservation or prevention of left ventricle function impairment and QoL improvement are the aims of rate control drugs. This treatment should prevent tachycardiomyopathy and the development of HF. In order to maintain an adequate cardiac output, lower physiological demands, and prevent consequences, an appropriate ventricular rate should be achieved. If the ventricular rate is very rapid or slow, it could cause undesired adverse effects (e.g. pacemaker implantation, impaired QoL, and higher cost) [30].

However, in response to AF, ventricular rates have to increase in order to maintain homeostasis in the cardiovascular system due to the atrial conduction system being impaired and not contracting properly. Moreover, appropriate ventricular rates may differ from patient to patient. For instance, HF preserve ejection fraction (HFpEF) often requires slower ventricular rates to allow for more diastolic filling time [22]. However, American guidelines (American Heart Association/American College of Cardiology/Heart Rhythm Society) for the management of AF are more inclined to maintain a strict rate strategy and heart rate (defined as a ventricle rate <80 bpm at rest) in symptomatic patients [27].

The lenient strategy (defined as a ventricle rate <110 bpm at rest) could be reasonable in asymptomatic patients who have preserved left ventricle systolic function. Also, the optimal ventricle rate control in individuals with AF and HF with either preserved or reduced ejection fraction is unclear. The European Society of Cardiology for heart failure guidelines (2020) recommends a relatively lenient rate control strategy (60–100 beat per minutes at rest) in AF patients with HF [2]. In contrast, a ventricular rate < 80–90 bpm at rest and < 110–130 bpm during moderate-intensity exercise is supported by the ACC/AHA HF guideline [31]. Nevertheless, regardless of the recommendations in these guidelines, the main priority is based on the clinical decision.

Using the rate control strategy requires a comprehensive evaluation of AF patterns/types (e.g. paroxysmal, persistence, long standing persistence or permanent), symptoms, physical activity level, underlying diseases, age, assessment of cardiac functions and reconsideration of AF ablation. In some circumstances, a high heart rate is often necessary to maintain the physiological demands of physical activity and to prevent development of HF [32]. HFrEF and slow heart rates have been associated with higher mortality [32,33]. Often, symptoms such as fatigue and exercise intolerance may be due to chronotropic incompetence or relative bradycardia, such as bradycardia-tachycardia syndrome. Thereby, titrating rate control drugs should be initiated with or without the implementation of a pacemaker. To avoid the development of tachycardiomyopathy or persistent symptoms, increasing the rate control dosage may be necessary. Generally, no single regimen can exhibit a suitable approach to obtain an effective treatment in all AF patients, but it is worth noting that the lenient pharmacologic rate approach is convenient, safe and effective in a wide range of individuals and must be deemed as a first-line treatment.

### Pharmacological rate control approaches

The purpose of the rate control approach is to closely monitor and assess the patient condition before establishing any treatments. It has been reported that a rapid ventricular rate could exacerbate the underlying disease which led to different pharmacological or non-pharmacological managements than the standard management of AF. The ventricular rate in AF is mediated by sympathetic and parasympathetic activity and dromotropic effect produced by the atrioventricular (AV) node. In order to slow the ventricular rate in patients with AF, there are three common classes of drugs that can be used for the rate control approach: BB, non- dihydropyridine calcium channel blocker (NDCC) and, to a certain extent, digoxin or amiodarone.

Several rhythm and rate control agents have been linked to a higher risk of mortality in patient with HF (e.g. dronedarone) [34] or structural heart disease (e.g. flecainide, propafenone and d-sotalol) [35]. So, adverse events, contraindications and non-invasive multimodality imaging are important considerations that provide all needed information in the selection of rhythm and rate control agents [36]. Also, based on the currently available evidence from RCTs, the primary indication for rhythm control is to reduce AF-related symptoms and improve QoL [2]. Thus, if symptoms are not AF related then rate control agents should be used instead of rhythm control agents. Similar to the selection between rate and rhythm control, the decision of single or concomitant use among rate control drugs is based on symptoms, potential adverse events, and the presence of comorbidities (e.g. HFpEF, HFrEF, severe chronic obstructive pulmonary disease (COPD) or asthma). In the recent European Society of Cardiology (ESC) guidelines on the management of AF, BB are the first-line treatment for those with HF regardless of their LV function/status (i.e. HFrEF or HFpEF) [2], and calcium channel blockers can be used in those with preserved ejection fraction and severe COPD and asthma [2]. However, in cases of suboptimal rate control (resting heart rate >110 bpm), worsening of symptoms or QoL, consider second-line treatments such as digoxin, and, if necessary, third-line treatments option of a combination of three drugs, or evaluation for CRT-P, CRT-D or AVN ablation and pacemaker implantation should be considered.

Beta blockers (BB)are sympatholytic agents that inhibit the activity of the beta-1 receptor in the AV node and thereby reduce accelerated ventricular rate. A randomized, double-blind study [37] in patients with persistent or permanent AF who were using the beta blocker carvedilol showed a significant graded reduction in heart rate with each dose up-titrating from 5 to 20 mg, which suggests a trend for dose-dependent heart rate reduction. Carvedilol is recommended for patients with HF and reduced LVEF because large randomized controlled trials have demonstrated a substantial reduction in morbidity and mortality rates in patient assigned to BB [38–40]. However, this result was not found in patients with AF [41]. This may be due to the large reduction in ventricle rate in AF patients. It is possible that a lower dose of BB applied to a faster heart rate could be beneficial [32–33]. It has been shown that BB have the advantage of improving the LVEF in patients with chronic HF. According to a retrospective analysis [42], in patients with established AF and chronic HF, carvedilol therapy showed a statistically significant improvement in LVEF and a potential decrease in the combined endpoint of death or hospitalization. However, the study had a sample size with more than 90% of the subjects on digoxin in both arms, which does not allow for determining which benefits resulted from carvedilol or digoxin. Nevertheless, this retrospective study shed light on subsequent studies of AF with HF, despite the prognostic benefit of BB seen in patients with HFrEF and sinus rhythm being questioned in patients with AF and HF [41].

Non-dihydropyridine calcium channel blockers (NDCC), such as verapamil or diltiazem, slow the conduction of the AV node and have negative inotropic and chronotropic effects. In AF patients, NDCC produce acceptable rate control levels [43], but it is better to avoid the use of NDCC in patients with HFrEF due to their negative inotropic effects [44]. NDCC can improve arrhythmia-related symptoms and exercise capacity as well as reduce the level of N-terminal prohormone brain natriuretic peptide (NT-proBNP; a marker for poor cardiac function) when compared with the use of BB, as demonstrated in a small trial of low-risk patients with preserved LVEF [45].

Digoxin reduces ventricular rate during AF by slowing AV nodal conduction but has no direct effect on the ventricles. It is not recommended for patients with high sympathetic activity (e.g. hyperthyroidism) [27]. In a recent study, it was demonstrated that verapamil significantly increased the concentration of digoxin levels in patients who simultaneously received digoxin, but this trend appears to be dependent on the digoxin dosage [46]. Therefore, low doses of digoxin in elderly patients combined with medication that elevates digoxin serum concentrations should be used with caution [47].

Several studies have called for digoxin safety in AF patients, yet whether these safety issues result from patient’s comorbidities or the drug itself is unclear. According to a retrospective analysis of a digoxin study [48], higher digoxin concentrations were shown to be associated with higher mortality rates in HF patients. The AFFIRM trial [49], which was conducted on patients with AF, showed that digoxin was independently associated with higher mortality. However, two post-hoc analyses of the AFFIRM database showed conflicting results on cardiovascular outcomes for AF patients using digoxin. For example, digoxin was independently associated with a 41% increased risk of death, regardless of HF status [50].

Another study showed that digoxin did not have a significant effect on mortality [51]. Even though the same database was used, different statistical analyses have resulted in different conclusions. These different conclusions were attributed to the differences in chosen study populations or exposure classification (i.e. fixed vs. time-varying) as differences in analytical techniques [52].

A retrospective analysis confirmed an increased risk of death with digoxin in AF patients [53], but a meta-analysis in patients with AF with or without HF showed a normal effect on mortality and all-cause hospital admission [54,55]

It is difficult to attribute poor outcomes to digoxin itself without taking into consideration the patient’s comorbidities and other treatment failures. Due to the lack of studies that support digoxin’s effectiveness during exercise as a rate control therapy, the use of digoxin has declined [56]. A systematic review with meta-analysis and trial sequential analysis showed that, based on current available evidence, the clinical effects of digoxin on serious adverse events, QoL, HF, stroke, and all-cause mortality are uncertain [57]. In reducing heart rate, digoxin appears to be superior compared with a placebo, but inferior compared with BB [57]. Despite this, in patients with AF and HF, the concomitant use of digoxin and carvedilol appears to be superior compared to using either drug alone [56]. The RATE-AF trial [58] compared two rate control strategies (digoxin vs. the beta blocker bisoprolol). This trial showed that digoxin therapy has a similar effect as BB on reducing heart rate and QoL in older patient with permanent AF and symptoms of HF. However, digoxin was associated with a higher reduction in natriuretic peptides and adverse events, and an improvement in some measures of QoL compared with BB. However, on the basis of this finding from a small, open-label in design, the clinical practice guidelines for rate control may not substantially changed; thus, digoxin may be favoured as a second-line therapy for those with permanent AF and intolerant or inadequate response to BB or NDCC [58].

Sotalol is a combination of BB with potassium channel blockers (Class III antiarrhythmic effect) [59] that slows the conduction velocity (i.e. dromotropic effect) [60] and can prolong the QT interval by blockading the rapid components of delayed rectifier potassium current. These effects are essential for cardiac action potential (i.e. the repolarization of phase 3) [61,62], but may cause a *torsades de pointes* as demonstrated in the PAFAC trials [63,64]. In post-myocardial infarction patients with left ventricle dysfunction, sotalol was associated with a higher mortality compared to the placebo group, which is probably because of ventricular arrhythmias [65,66]. However, in two controlled trials, there were no potential risks found as a result of the use of sotalol [67,68]. Overall, as a rate control strategy, sotalol is not recommended [69,70]. Some class III antiarrhythmic drugs have shown reverse use dependence (i.e. inverse correlation between heart rate and QT interval) on action potential duration, as seen with sotalol [61]. This means as the heart rate slows, the QT interval can be prolonged, which may elucidate the association between bradycardia and *torsades de pointes*, resulting in the ineffectiveness of sotalol for significant tachycardia.

Sotalol has a BB-like effect extending sinoatrial cycle length (i.e. reduced heart rate), decreasing AV node conduction, and increasing AV node refractoriness (i.e. PR interval prolongation) [71]. Since sotalol has rhythm control and rate control properties, it is indicated as sinus rhythm maintenance for post-cardioversion or post-cardiac surgery in AF patients with underlying coronary heart disease. A meta-analysis showed that compared to a placebo or even BB, sotalol was substantially more effect for the prevention of AF, and it had a similar effect to amiodarone [72]. In the DAPHNE trial, 135 patients with bradycardia-tachycardia syndrome were randomly assigned to sotalol or either of the beta receptor blockades metoprolol or atenolol one month after treatment using a rate-adaptive dual-chamber pacemaker [73]. As a result, almost 30% of patients were free from atrial tachyarrhythmia recurrences in both the beta blocker and sotalol group, and the rates of cardioversion and hospitalization in the sotalol or BB groups were not significantly different for either group. Survival analysis demonstrated a trend towards a lower incidence of cardioversion or hospitalization among the beta blocker group. In comparison with other antiarrhythmic medications, sotalol seems to be less effective, either when administrated orally [74] or intravenously [75,76]. Similarly, dronedarone slows the conduction velocity as a rate control drug [77]; however, in patients with permanent AF, it increases the risk of cardiovascular morbidity (i.e. stroke and HF) and mortality. For this reason, it is contraindicated for this group [34]. Lastly, the class III antiarrhythmic drugs (class III) have rate-control properties (e.g. sotalol, dronedarone, and amiodarone), but they should only be used for rhythm control, whereas class IA and IC antiarrhythmic drugs have no role in rate control and may paradoxically increase heart rate by reducing atrial rate.

### Non-pharmacological rate control approaches

When pharmacological rate control medication fails, ablate and pace strategy can control ventricular rate with atrioventricular nodal (AVN) ablation and implantation of a pacemaker. It is a relatively simple procedure with low rate of complication [78], such as worsening left ventricle ejection fraction [79], and may even improve left ventricle ejection fraction in selected patients [80]. The timing of pacemaker implantation plays a major role in lowering the risk of long-term mortality, when the pacemaker implanted few weeks prior to AVN ablation and set at a pace rate of 70–90 bpm [81]. The selection of pacemaker type or pacing mode is still unclear; however, patient characteristics determine the choice of pacing therapy whether right or bi-ventricle pacing [82], although there are limited data on the advantage of RV vs. bi-ventricle pacing in HF patients. Interestingly, in a small RCT, in patients with permanent AF and who have severe symptoms, it was shown that ablate and cardiac resynchronized therapy was superior to pharmacological rate control agents in symptom relief and reducing HF hospitalization and mortality [83].Thus, atrioventricular nodal ablation in AF helps in limiting the rapid ventricular rate from aggravation symptoms and HF. Growing evidence suggests that His-bundle pacing, an attractive alternative pacing mode, could be useful in severely symptomatic patients with permanent AF [84], and it is being tested in a current ongoing clinical trial (NCT02805465).

### Rate Control medications in different patient groups

Several studies have examined different medications with the aim of managing acute or chronic AF, but unfortunately the limitations of these trials, such as short-term follow-up and a small number of patients, have resulted in low-quality evidence. In acute care settings for AF, pharmacological rate control is recommended for patients who experience moderate symptoms or hemodynamic distress. However, it is recommended that the heart rate should not exceeds 100 bpm, but the exact optimal heart rate has still not properly been defined, and therapy is often symptom-guided. In acute settings of HF with reduced LVEF, amiodarone can be considered as a good alternative compared to BB and NDCC [85].

For chronic AF, no robust recommendations for a specific rate control drug have been made. The determinant of therapy relies on the pre-existence of comorbidities and HF. In a small study of 12 patients with permanent AF, the subjects underwent five different drug regimens over a two-week period. These drugs included atenolol (50 mg), diltiazem-CD (240 mg), digoxin (0.25 mg), digoxin plus diltiazem and lastly digoxin plus atenolol. Digoxin plus atenolol was the most effective rate control drug as it yielded the lowest ventricular rate during exercise; digoxin alone and diltiazem alone were the least efficient regimens [86]. The randomized study, RATAF [43], compared the effects of four rate-reducing drugs (metoprolol 100 mg/day, carvedilol 25 mg/day, verapamil 240 mg/day and diltiazem 360 mg/day) on ventricular rate and symptoms in permanent AF patients without reduced LVEF or HF. The results showed that diltiazem was the most effective at reducing heart rates, and both NDCC agents (verapamil and diltiazem) reduced the symptoms related to arrhythmia and improved exercise tolerance. Conversely, both BB were less effective on exercise tolerance, ventricular rate, and arrhythmia-related symptoms. RATAF II is an ongoing Phase 4 trial designed to compare the effects of two rate control drugs in patients with permanent AF, hypothesizing that a 6-month treatment with the NDCC diltiazem will lower the level of NT-pro BNP and increase exercise capacity (peak VO_2_; volume oxygen) compared to treatment with the beta blocker metoprolol, as low levels of NT-pro BNP are associated with sinus rhythm maintenance. The RATAF II trial may encourage more NDCC prescriptions if it yields positive outcomes (NCT02695992).

Moreover, patients with recent-onset AF could benefit from a wait-and-watch approach with the use of rate control agents only (i.e. beta blocking agents, non-dihydropyridine CCBs, or digoxin) and delayed cardioversion if needed within 2 days of symptom onset in paroxysmal AF. This wait-and-watch strategy was safe and non-inferior to the immediate cardioversion at4 weeks [87]. It also shows that using rate control agents instead reduces the need for cardioversion (whether pharmacological or electrical) and allows for spontaneous conversion, as frequently occurred in the delayed cardioversion group [87]. However, this specific rate control strategy has not been extensively studied in terms of the selection between the rate control agents. In observation study in acute AF patients, the clinical determinants of early spontaneous conversion were most likely recent-onset, short-duration AF episodes, lower BMI, and normal size of left atrium [88].

### Rate control monitoring

Even though no specific recommendations suggest the necessity to monitor patients on rate control drugs, assessments of ventricular rates while resting and during exercise are highly recommended. It is necessary to assess ventricular rates during moderate exercise, since rapid increases in ventricular rate are accompanied by symptoms at rest that require strict rate control along with a 24-hour Holter monitor to assess safety concerns [89]. Adequate heart rate can be determined by an exercise test or remote monitoring devices.

For rate control monitoring, 12-lead ECG can only provide a time-point snapshot of cardiac rhythm in the clinical setting, while remote monitoring devices, such as (ZioPatch, AliveCor) have emerged as some of non-invasive AF detection and monitoring.

ZioPatch, an adhesive patch, is a single-lead ECG monitor provides a continuous monitoring for up to 14 days, and FDA approved [90], and allow the patients to perform their usual daily activities [91]. Several clinical trials have shown that the ZioPatch accurately identifies more episodes of AF compared to two-days ambulatory ECG device and high patient adherence with the device [91,92]. Thus, referred patients with AF symptoms such as, palpitations or syncope that often occurs more than 14-days period would more benefit from this way [92].

Similarly, AliveCor, mobile ECG recorder, that is FDA-approved (uses smartphone app) to capture arrythmias, including episodes of AF [93], with high sensitivity and specificity (94% and 98%; respectively). Studies have shown that the AliveCor is beneficial for detecting AF and it is easy to use [93,94], but this was based on case reports which successfully detect the recurrent episodes, however ongoing iHEART trial will evaluate the utility in recent-onset AF patient for larger scale in real world settings [95]. It will shed the light in the ability of this device for detection and treatment of recurrent AF, eventually improve the overall monitoring and management of atrial fibrillation. Thus, frequent and long episodes could be better captured by AliveCor, in contrast, shorter-time episode may be less suited for AliveCor.

Nevertheless, multimodalities imaging can offer a valuable information for the structural, functional and anatomical changes of both atrial and ventricles. A transthoracic echocardiogram (TTE) provides an evaluation of function and size of atrial and ventricles, such as valvular heart disease, LV hypertrophy, and most importantly LA appendages thrombus prior sinus rhythm restoration. CT coronary angiography assess the coronary heart disease, and the brain CT and MRI provides more insight in suspected stroke [96]. Thus, the decision between or among rate and rhythm control will depend on the functional and structural state of the heart as these imaging modalities provides an insightful information.

## Conclusions

In summary, the rate control approach (i.e. maintaining an adequate ventricular rate for hemodynamic stability, haemostasis and prevention of serious adverse effects) is the first-line treatment for AF management and frequently adequate for improving AF-related symptoms. There is insufficient high-quality evidence to inform the intensity and type of rate control therapy. A lenient rate control strategy is convenient, safe, and extends across a wide spectrum of AF patients. It should be used as an initial therapy for patients with a good response to lenient rate control and who have mild symptoms. If symptoms persist or the left cardiac function begins to deteriorate, a strict rate control treatment should be considered. Special consideration is required for those with bradycardia-tachycardia syndrome, new-onset AF, and implantable cardioverter defibrillator. The optimal ventricular rates at rest and during exercise remain undetermined. Therefore, although rate control drugs are the initial management recommendation for all AF cases, a number of issues remain to be solved.
